# CRMP4 and CRMP2 Interact to Coordinate Cytoskeleton Dynamics, Regulating Growth Cone Development and Axon Elongation

**DOI:** 10.1155/2015/947423

**Published:** 2015-05-10

**Authors:** Minghui Tan, Caihui Cha, Yongheng Ye, Jifeng Zhang, Sumei Li, Fengming Wu, Sitang Gong, Guoqing Guo

**Affiliations:** ^1^Department of Anatomy, Medical College of Jinan University, Guangzhou 510630, China; ^2^Postdoctoral Stations of Integrated Traditional and Western Medicine, Medical College of Jinan University, Guangzhou 510630, China; ^3^Department of Pediatrics, The First Affiliated Hospital of Jinan University, Guangzhou 510630, China; ^4^Department of Pediatrics, Guangzhou Women and Children's Medical Center, The Affiliated Hospital of Guangzhou Medical University, Guangzhou 510120, China; ^5^Department of Pathophysiology, Medical College of Jinan University, Guangzhou 510630, China

## Abstract

Cytoskeleton dynamics are critical phenomena that underpin many fundamental cellular processes. Collapsin response mediator proteins (CRMPs) are highly expressed in the developing nervous system, mediating growth cone guidance, neuronal polarity, and axonal elongation. However, whether and how CRMPs associate with microtubules and actin coordinated cytoskeletal dynamics remain unknown. In this study, we demonstrated that CRMP2 and CRMP4 interacted with tubulin and actin *in vitro* and colocalized with the cytoskeleton in the transition-zone in developing growth cones. CRMP2 and CRMP4 also interacted with one another coordinately to promote growth cone development and axonal elongation. Genetic silencing of CRMP2 enhanced, whereas overexpression of CRMP2 suppressed, the inhibitory effects of CRMP4 knockdown on axonal development. In addition, knockdown of CRMP2 or overexpression of truncated CRMP2 reversed the promoting effect of CRMP4. With the overexpression of truncated CRMP2 or CRMP4 lacking the cytoskeleton interaction domain, the promoting effect of CRMP was suppressed. These data suggest a model in which CRMP2 and CRMP4 form complexes to bridge microtubules and actin and thus work cooperatively to regulate growth cone development and axonal elongation.

## 1. Introduction

Proper axonal elongation and path finding are critical for neurons to reach their destination and form accurate neuronal circuits. Extracellular developmental guidance stimulators, such as growth factors, cell adhesion molecules, and other cues, are responsible for navigating the growth cones of an extending axon through the modulation of the cytoskeleton, which includes changes produced by actin and microtubules [1, 2]. Numerous studies in recent decades elucidated the role of actin and microtubule dynamics separately in axonal guidance and growth cone development [3–5]. However, the coordination of microtubule and actin dynamics is more essential than their separate involvement in axonal guidance, and detailed mechanisms showing how they achieve such cooperation are still emerging [6].

Collapsin response mediator proteins (CRMPs), consisting of five cytosolic proteins (CRMP1–CRMP5), are a family of proteins that are highly expressed in developing and adult nervous systems [7–9]. CRMPs function in a variety of cellular processes, including cell migration, differentiation, neurite extension, and axonal regeneration [10, 11]. Unlike microtubule-associated proteins (MAPs), CRMPs likely exist as homo- or heterotetramers* in vivo* [12], do not have enzymatic activities, and are regulated by phosphorylation [13, 14]. The final target of the CRMPs is the cytoskeleton [15, 16]. For example, CRMP2 regulates axonal growth and neuronal polarity [17] by promoting microtubule assembly and stability [18]. CRMP5 interacts with tubulin to inhibit neurite outgrowth by modulating CRMP2 [19]. CRMP4 regulates actin cytoskeleton in neuroblastoma cells to promote cell migration [16]. However, whether and how CRMPs associate with microtubules and actin coordinated cytoskeletal dynamics remain unknown.

In this study, we demonstrate that CRMP2 and CRMP4 interact with both microtubules and actin in growth cone lysates. CRMP2 and CRMP4 each colocalize with tubulin and actin at the transition- (T-) zone in growth cones. In addition, CRMP2 and CRMP4 interact not only with the cytoskeleton but also with one another to form complexes and function coordinately to regulate axonal elongation by modulating their interaction with cytoskeleton. A model of CRMP2- and CRMP4-mediated coordination of microtubule and actin dynamics in growth cone development and axonal elongation is presented.

## 2. Materials and Methods

### 2.1. Animals

The experiments were conducted with 1-day-old Sprague Dawley rats. All animal procedures were performed in strict accordance with the recommendations in the* Guide for the Care and Use of Laboratory Animals* produced by the National Institutes of Health. The protocol was approved by the Institutional Animal Care and Use Committee at Jinan University. All efforts were made to minimize the suffering and number of animals used.

### 2.2. Growth Cone Purification

Growth cone purification was performed based on methods described in previous reports [20, 21]. Briefly, hippocampi from brains were dissected from fetal rats at 18 days of gestation and homogenized using a Teflon-glass homogenizer in approximately eight volumes (w/v) of 0.32 M sucrose containing 1 mM MgC1_2_, 1 mM TES-NaOH, pH 7.3, and the following protease inhibitors: 3 mM aprotinin (Calbiochem, San Diego, CA), 20 mM benzamidine, 1 mM leupeptin, 1 mM pepstatin A, and 0.6 mM phenylmethylsulfonyl fluoride (all from Sigma, St. Louis, MO, USA). The homogenate was centrifuged at 1,300 rpm for 15 min. The obtained supernatant was loaded onto a discontinuous sucrose density gradient consisting of three layers: 0.75, 1.0, and 2.66 M. The density gradients were centrifuged to equilibrium at 35,000 rpm for 200 min in a Beckman SW40Ti vertical rotor (Beckman Instruments, Palo Alto, CA). The A-fraction containing the growth cones was collected for further analysis.

### 2.3. Cell Culture and Transfection

Hippocampi were dissected from postnatal rat pups (days 0 to 1, Sprague Dawley), and dissociated hippocampal neurons were obtained using 0.125% trypsin and plated at a density of 1 × 10^4^cells/cm^2^ onto poly-D-lysine-coated glass coverslips. Cultures were maintained in Neurobasal-A medium containing 2% B27 and 0.5 mM glutamine supplemented at 37°C in a 5% CO_2_ humidified incubator (Thermo, USA). Half of the culture media was replaced every 3 days. Calcium phosphate transfections with various constructs were conducted on 1 day* in vitro* (DIV). Experiments for growth cone observation were performed, 2 DIV and for axonal growth 4 DIV. Human embryonic kidney (HEK) 293 cells were maintained in Dulbecco's modified Eagle's medium supplemented with 10% fetal bovine serum and penicillin/streptomycin (Invitrogen, California, USA) in a 5% CO_2_ incubator at 37°C. Calcium phosphate was used to transfect the constructs into the HEK293 cells. After transfection, cells were grown for 36–48 h before harvesting.

### 2.4. Plasmids and Constructs

The cDNA encoding the full-length rat CRMP4 was obtained using PCR according to a previously published method [22]. The full-length CRMP4 cDNA was inserted into the pcDNA3.1-V5/His-A vector using the same method as previously published for CRMP2 [23]. The cDNAs encoding rat CRMP2ΔC322 (amino acids 323–572 deleted) and CRMP4ΔC471 (amino acids 472–572 deleted) were amplified with PCR and subcloned into the pcDNA3.1-V5/His-A vector. The CRMPs or truncated CRMPs tagged with glutathione S-transferase (GST) at the N-terminus were generated by subcloning into the pGEX-4T-1 vector (Amersham Pharmacia Biotech, Buckinghamshire, UK). All constructs were verified by sequencing.

### 2.5. Recombinant Protein Expression and GST-Pulldown Assay

The GST fusion CRMP expression and pulldown assays were performed as previously described [24]. Briefly, GST-CRMP constructs were transformed into the BL21 (DE3) strain of* Escherichia coli* (Invitrogen). The production of fusion proteins was induced by incubation with 0.2 mmol/L isopropyl-1-thio-*β*-D-galactopyranoside for 6 h at 20°C. The bacteria were centrifuged and resuspended with a cocktail of protease inhibitors (Merck, Whitehouse Station, NJ). The cell suspension was treated with 0.1% lysozyme, followed by 0.5% deoxycholic acid on ice for 20 min. After sonication, the cell debris was removed by centrifugation (15,000 g for 30 min). The supernatant, with 1% Triton X-100, was used for the purification of the GST fusion proteins using glutathione-Sepharose beads.

### 2.6. Western Blotting

Western blot analysis was performed as described previously [25]. Briefly, lysates were separated using SDS-PAGE and electrophoretically transferred to a polyvinylidene difluoride membrane. Membranes were blocked in Tris-buffered saline with 5% milk and 0.05% Tween and probed with primary antibodies at 4°C overnight. Rabbit or mouse antibodies against CRMP2 and CRMP4 as well as a mouse anti-V5 antibody were purchased from Abcam (Cambridge, UK); mouse anti-actin and anti-tubulin were obtained from Sigma. After being washed, the membranes were incubated with horseradish peroxidase-conjugated goat anti-mouse or anti-rabbit secondary antibodies (Jackson ImmunoResearch, West Grove, PA) and visualized using enhanced chemiluminescence reagents.

### 2.7. Immunoprecipitation

Immunoprecipitation (IP) assays were performed as described previously [24]. For hippocampal neuron immunoprecipitation, neuronal extracts were prepared by solubilization in 400 *μ*L of cell lysis buffer for 10 min at 4°C. After a brief sonication, the lysates were centrifuged at 15,000 ×g for 10 min at 4°C. The cell extract was immunoprecipitated with 4 *μ*g of antibodies against CRMP2 (IBL-America, Gunma, Japan), CRMP4 (Abcam), and actin and tubulin (Sigma) and incubated with 60 *μ*L of protein G plus protein A agarose for 16 h at 4°C using continuous inversion. The immune complexes were pelleted and washed three times. The precipitated complexes were then subjected to western blot analysis.

### 2.8. Immunocytochemistry

Neurons were grown on coverslips and processed according to immunocytochemistry protocols described previously [23]. Hippocampal neurons were fixed using freshly prepared 4% paraformaldehyde, followed by permeabilization with 0.1% Triton X-100 in TBS and blocking in 3% normal donkey serum. The cells on the coverslips were incubated with the following primary antibodies: rabbit or mouse anti-CRMP2 or anti-CRMP4 (Abcam) and mouse anti-tubulin or anti-actin (Sigma). Rabbit primary antibodies were detected with an anti-rabbit secondary antibody conjugated to Alexa Fluor 488 (Molecular Probes, Leiden, Netherlands). The mouse primary antibodies were detected using an anti-mouse secondary antibody conjugated to Alexa Fluor 555 (Molecular Probes).

### 2.9. RNA Interference

A CRMP4 siRNA (siCRMP4) fragment (5′-GGCCGTTCTAACATCACAT-3′) and a scrambled sequence (negative control, NC) were synthesized by Shanghai GenePharma Co., Ltd. (Shanghai, China) using the Whitehead siRNA Selection Program [26]. To determine the efficacy and specificity of the siRNA, the NC or siCRMP4 was cotransfected with rat FLAG-CRMP4 plasmids into HEK293 cells. The expression of FLAG-CRMP4 protein was examined using western blot analysis with a FLAG antibody. Transfections of siRNA in hippocampal neurons were conducted using a calcium phosphate protocol [23]. A GFP expression plasmid was cotransfected with the siRNAs to mark the transfected cells. The transfection efficiency in neurons was determined by calculating the percentage of GFP-positive cells in the total cell number. The total number of neurons counted for each treatment group was more than 100.

### 2.10. Morphometry of Hippocampal Neurons

The morphology of the growth cones and axons from the transfected hippocampal neurons in primary cultures was analyzed using images of individual neurons randomly captured in a blinded manner with an Axio Observer Z1 microscope (Carl Zeiss, Oberkochen, Germany) using a 40x objective. The images were analyzed using Image-Pro Plus 6 software (Media Cybernetics). Images were measured in an unbiased manner and were scored blindly, that is, without previous knowledge of the treatments. More than 30 cells were examined in three independent experiments. For the colocalization analysis, neurons were scanned using a 63x oil immersion objective mounted on a confocal microscope (LSM 710 Meta; Carl Zeiss). Images were recorded with sequential acquisition settings at a resolution of 1024 × 1024 pixels and a 12-bit depth and processed using LSM 710 software. For normalization, the average area of the growth cones and the average axon length in the control group were set to 100%.

### 2.11. Statistical Analysis

All experiments were repeated at least three times using independent culture preparations, and the analyses were performed blindly. The statistical significance of the differences was analyzed using Student's *t*-test between two groups and one-way ANOVA with Newman-Keuls* post hoc* tests for comparisons among more than two groups.

## 3. Results

### 3.1. CRMP2 and CRMP4 Interact with the Cytoskeleton* In Vitro*

**Figure 1:**
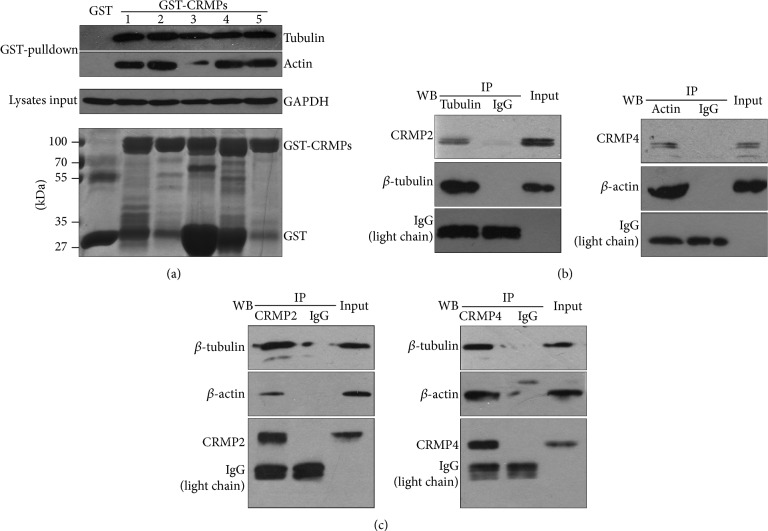
CRMP2 and CRMP4 interact with tubulin and actin. (a) Bacterial recombinant glutathione S-transferase- (GST-) CRMPs were purified and subjected to GST-pulldown assays with growth cone extracts from rat brain. Coomassie blue staining of GST and GST-CRMPs (bottom) showed equal loading of bait proteins. The pulldown sediments were subjected to western blot assays with tubulin and actin antibodies using GAPDH as the lysate input control. Each result is representative of three to five separate experiments with similar results. (b) Growth cone lysates from rat brain were subjected to coimmunoprecipitation (Co-IP) with tubulin or actin antibody and then processed for western blot (WB) analysis with the indicated antibodies. (c) Growth cones lysates were immunoprecipitated with CRMP2 or CRMP4 antibodies and processed for WB assays to detect the indicated proteins using the appropriate antibodies.

To determine whether CRMPs are involved in the coordinated movement of microtubules and actin in growth cones during axonal growth, the interaction of CRMPs with the cytoskeleton was examined. We first purified GST-CRMP proteins and baited them with the growth cone lysates derived from hippocampal neurons. CRMP1–CRMP5 showed nearly equal abilities to interact with tubulin and actin, except for CRMP3 that had less affinity for actin. By contrast, the GST control group showed no interaction with either tubulin or actin. The GAPDH control indicated equivalent loading for the various growth cone lysates, and Coomassie blue staining of GST and GST-CRMPs showed equal loading of the GST-tagged bait proteins (Figure 1(a)). To further confirm the association of the CRMPs with the cytoskeleton, a coimmunoprecipitation (Co-IP) assay was performed with rat growth cone extracts. Because the interactions of CRMP2 with tubulin [18] and CRMP4 with actin [16] were previously established, we chose these two proteins for the Co-IP assay. We found that CRMP2 was precipitated with the tubulin antibody, and CRMP4 interacted with actin (Figure 1(b)). Both tubulin and actin were precipitated using CRMP2 or CRMP4 antibodies (Figure 1(c)). These data suggest that CRMP2 and CRMP4 interact with the cytoskeleton* in vitro*.

### 3.2. Colocalization of CRMP2 and CRMP4 with the Cytoskeleton in Growth Cones

**Figure 2:**
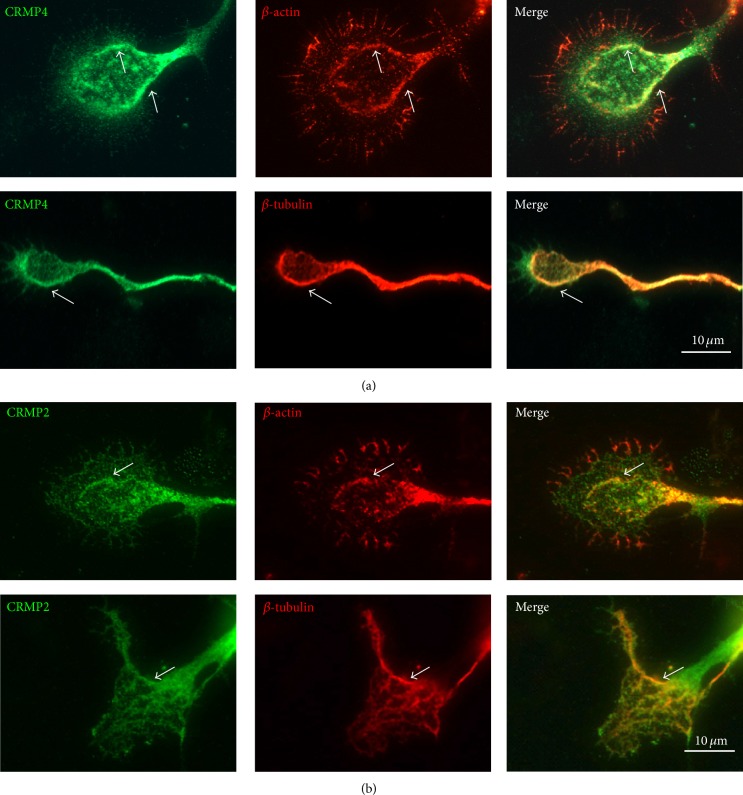
CRMP2 and CRMP4 colocalize with tubulin and actin in growth cones. (a) Anti-CRMP4, anti-tubulin, and anti-actin antibodies were used to detect endogenous proteins in the growth cones of hippocampal neurons. (b) Anti-CRMP2, anti-tubulin, and anti-actin antibodies were used to detect endogenous proteins in the growth cones of hippocampal neurons. Scale bar: 10 *μ*m.

To investigate how CRMP2 and CRMP4 interact with microtubules and actin in growth cones, the distribution of CRMP2 and CRMP4 was determined using immunofluorescence. We found that CRMP4 mainly distributed in the central (C) domain, with an especially strong signal in the transition- (T-) zone of the growth cone (Figure 2(a)). In addition, CRMP4 colocalized with microtubules and actin in the T-zone, where microtubules and actin filaments predominantly connect with one another [27]. CRMP2 showed the same localization pattern with microtubules and actin (Figure 2(b)). These results indicate that both CRMP2 and CRMP4 colocalize with the cytoskeleton.

### 3.3. CRMP2 and CRMP4 Interact with One Another* In Vitro*

**Figure 3:**
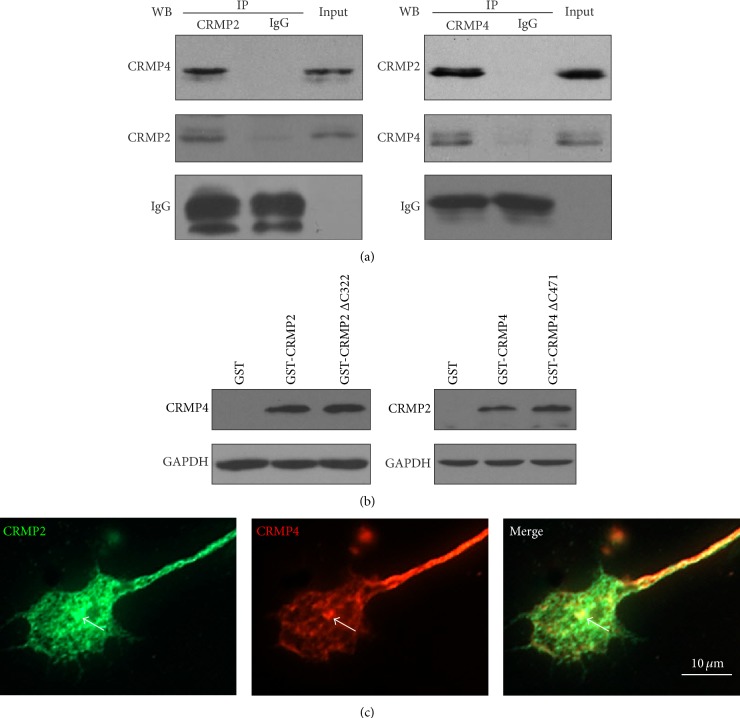
CRMP2 and CRMP4 form complexes in growth cones. (a) Growth cones lysates from rat brain were subjected to glutathione S-transferase- (GST-) pulldown assays using GST-CRMP2 or GST-CRMP4. The retrieved sediments were subjected to western blot analysis using the CRMP4 or CRMP2 antibodies. The GAPDH antibody was used to show equal loading. (b) Growth cones lysates from rat brain were subjected to coimmunoprecipitation (Co-IP) assays with rabbit CRMP2 and CRMP4 antibodies, and the resulting sediments were subjected to western blot analysis with mouse CRMP4 or CRMP2 antibodies. (c) Rabbit anti-CRMP2 and mouse anti-CRMP4 antibodies were used to detect endogenous proteins in the growth cones of hippocampal neurons. Scale bar: 10 *μ*m.

CRMPs can form homo- or heterotetramers with single or multiple isoforms [12, 28]. Therefore, we next asked whether CRMP2 and CRMP4 formed complexes with one another. Using a Co-IP assay, we found that CRMP4 was detected in the precipitated sediment of the CRMP2 antibody and CRMP2 was detected in the precipitated sediment of the CRMP4 antibody (Figure 3(a)), suggesting a potential* in vivo* interaction between CRMP2 and CRMP4. The core region, which lacks the C-terminal residues of CRMPs, was reported to be sufficient for tetramerization [12, 29, 30]. Therefore, we next constructed and examined two GST fusion plasmids, GST-CRMP2ΔC322 (CRMP2 1–322 AA) and GST-CRMP4ΔC471 (CRMP4 1–471 AA). The CRMP4 signal was detected in the GST-CRMP2 and truncated GST-CRMP2ΔC322 pulldown sediment, and, conversely, CRMP2 was detected in the GST-CRMP4/ΔC471 sediment (Figure 3(b)). We then determined whether either CRMP2 or CRMP4 was colocalized in the growth cones of hippocampal neurons. By applying immunocytochemistry methods, we detected CRMP2 and CRMP4 colocalized in the growth cones (Figure 3(c)). These results suggest that CRMP2 forms complexes with CRMP4 to interact with tubulin and actin in the growth cones of hippocampal neurons. These CRMP2/CRMP4 complexes may be the structural linkage between microtubules and actin.

### 3.4. Inhibition of CRMP2 and CRMP4 Impairs Axonal Development

**Figure 4:**
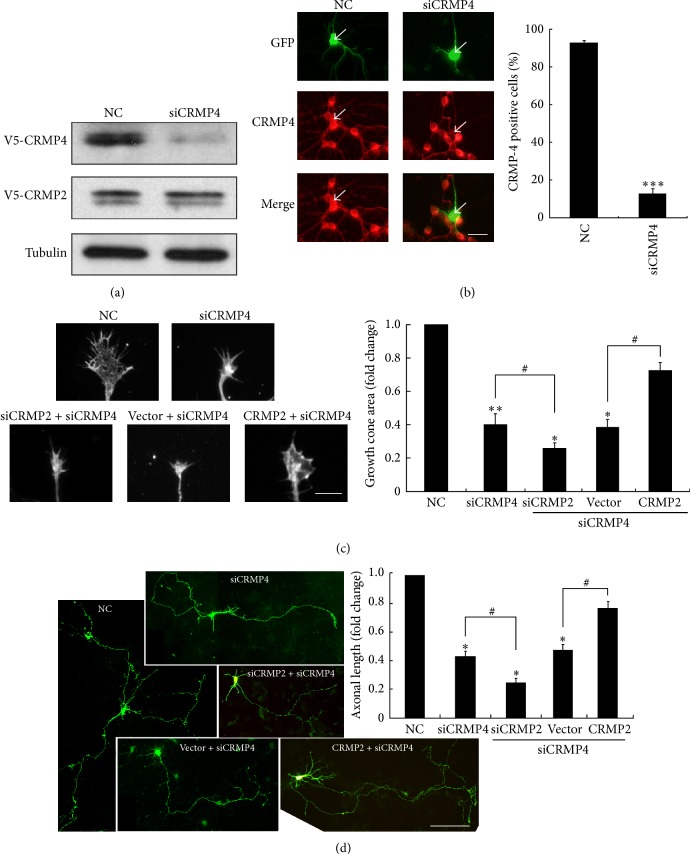
Both CRMP2 and CRMP4 are necessary for growth cone development and axonal elongation, and CRMP2 rescues the inhibitory effect of CRMP4 knockdown. (a) The efficiency of CRMP2 siRNA was measured in HEK293 cells by transfection with a nontargeting siRNA (negative control, NC) or the siCRMP4 fragment together with the CRMP4-V5 plasmid. Cell lysates of HEK293 cells were analyzed using western blotting with the V5 antibody. Tubulin was used as a loading control. (b) Neurons at 1 day* in vitro* (DIV) were transfected with GFP together with the CRMP4 siRNA fragments or NC. Neurons were stained with anti-GFP (green) and anti-CRMP4 (red). Representative images are shown (left panel). The percentage of CRMP4-positive GFP-transfected neurons (right panel). Mean ± SEM, *n* = 3; ^∗∗∗^ *P* < 0.001 versus NC. (c) Representative images of growth cones from neurons of the indicated transfections. Scale bar: 10 *μ*m. The relative ratio of the growth cone area of transfected cells was measured and is shown in the right panel. Mean ± SEM, *n* = 3; ^∗^ *P* < 0.05 versus NC; ^#^ *P* < 0.05 versus indicated control; ^∗∗^ *P* < 0.01 versus NC. (d) Hippocampal neurons were transfected with indicated plasmids or siRNA fragments together with GFP. Neurons were fixed at 4 DIV and stained with a GFP antibody. Axon length was measured as the mean ± SEM from three independent experiments. ^∗^ *P* < 0.05 versus NC; ^#^ *P* < 0.05 versus indicated control. Scale bar: 100 *μ*m.

Next, we asked whether this linkage mediated cytoskeletal dynamics to regulate growth cone development and axonal growth. The forced overexpression of CRMP4 was previously reported to increase axonal extension in hippocampal neurons [31]. However, how CRMP2 and CRMP4 work together to regulate axonal growth was not fully elucidated. Thus, we first investigated the effect of CRMP4 genetic silencing on growth cone development and axonal growth. We designed a siRNA fragment against CRMP4 using Whitehead siRNA Selection Web tools [26]. Either V5-CRMP2 or V5-CRMP4 expression plasmid was transfected into HEK293 cells along with the siCRMP4 fragment. The knockdown efficiency and specificity were determined using western blotting methods with a V5 antibody. As shown in Figure 4(a), the level of CRMP4 but not CRMP2 was significantly suppressed. In hippocampal neurons, the endogenous level of CRMP4 was also markedly silenced as shown by immunostaining (Figure 4(b)). Thus, both results showed the excellent silencing effect of the siCRMP4 fragment. The knockdown of CRMP4 dramatically reduced the size of the growth cones in hippocampal neurons at 3 DIV, and cosilencing with CRMP2 (using a fragment that was previously confirmed [23]) resulted in further growth cone shrinkage (Figure 4(c)). Overexpression of CRMP2 rescued the inhibitory effect of the CRMP4 knockdown on growth cone development (Figure 4(c)). Moreover, knockdown of CRMP4 impaired axonal growth, and cosilencing of CRMP2 caused further impairment. Overexpression of CRMP2 also partially rescued the inhibitory effect of CRMP4 siRNA on axonal growth, but the level remained less than that observed in the control (Figure 4(d)). These data indicate that CRMP4 is necessary and acts as a cofactor of CRMP2 to mediate growth cone development and axonal growth in hippocampal neurons.

### 3.5. Genetic Knockdown of CRMP2 Inhibits the Promoting Effect of CRMP4 on Axonal Development

**Figure 5:**
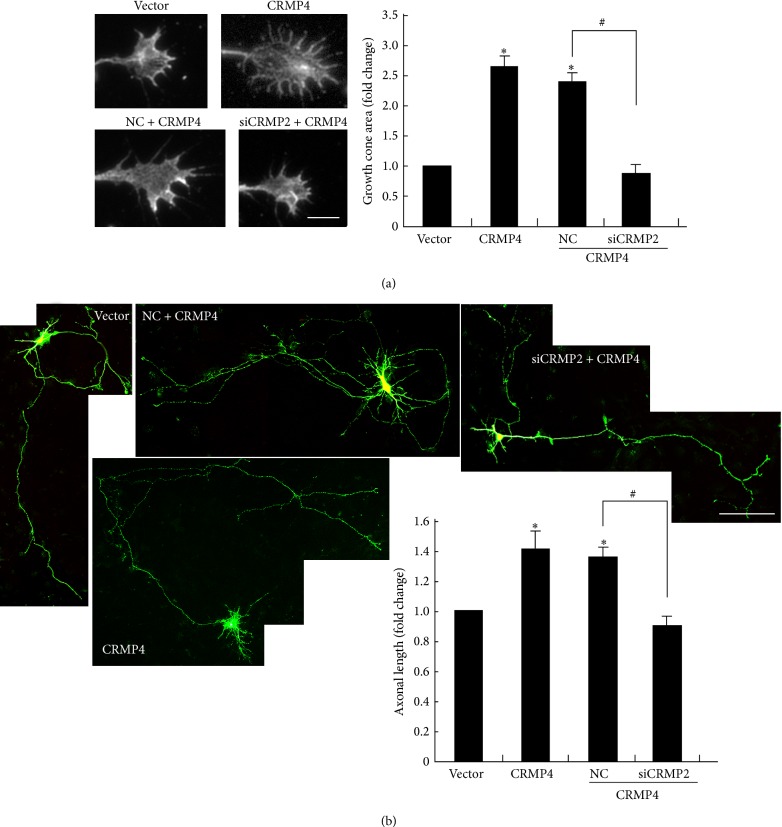
Genetic silencing of CRMP2 inhibits the promoting effect of CRMP4 on growth cone development and axonal elongation. (a) Representative images of growth cones with indicated transfections are shown. Scale bar: 10 *μ*m. Relative ratio of the growth cone area was measured and shown in the right panel. Mean ± SEM, *n* = 3; ^∗^ *P* < 0.05 versus vector control; ^#^ *P* < 0.05 versus NC. (b) Hippocampal neurons were transfected with the indicated plasmids or siRNA fragments together with GFP. Representative images of GFP staining are shown. Axon length was measured as the mean ± SEM from three independent experiments (*n* = 3). ^∗^ *P* < 0.05 versus vector control; ^#^ *P* < 0.05 versus NC. Scale bar: 100 *μ*m.

To further explore the relationship of CRMP2 with CRMP4-mediated axonal development, we asked whether CRMP2 knockdown could inhibit the promoting effect of CRMP4. As shown in Figure 5(a), we found that overexpression of CRMP4 markedly enlarged the size of the growth cones; however, when CRMP4 was cotransfected with the CRMP2 siRNA fragment, the size of the growth cones was not different from that of controls. We also found that overexpression of CRMP4 promoted hippocampal axonal growth, consistent with the results of a previous study [31]. Cotransfection of the siCRMP2 fragment blocked the promoting effect of CRMP4 on axonal growth (Figure 5(b)). These results suggest that CRMP2 and CRMP4 function collaboratively to regulate growth cone development and axonal elongation in hippocampal neurons.

### 3.6. CRMP2 and CRMP4 Promote Axonal Growth through Interaction with the Cytoskeleton

**Figure 6:**
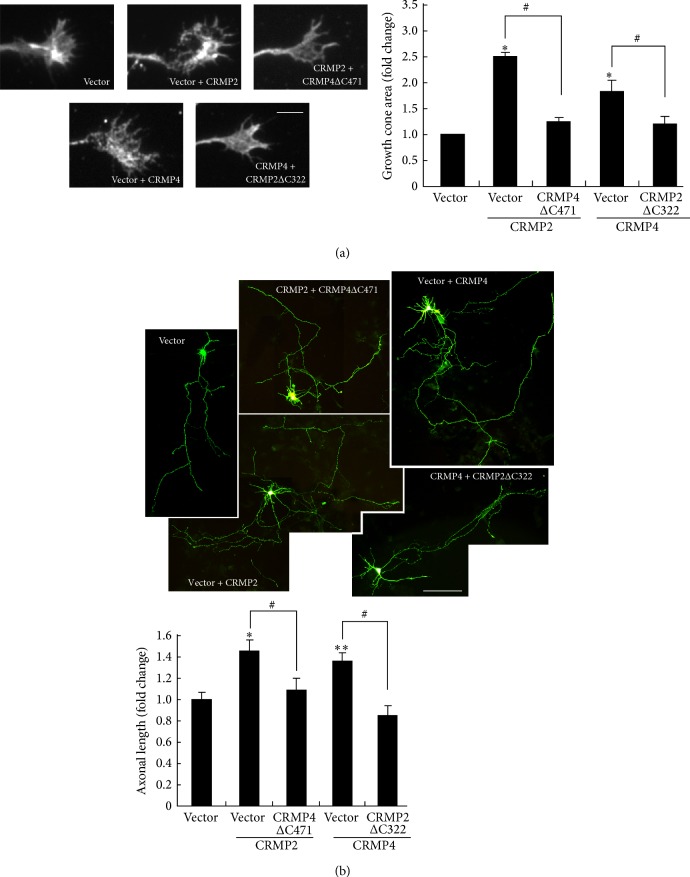
CRMP2 and CRMP4 interact with the cytoskeleton to promote growth cone development and axonal elongation. (a) Representative images of growth cones from neurons transfected with CRMP4 alone or together with CRMP2ΔC322 or with CRMP2 alone or together with CRMP4ΔC472 are shown. Scale bar: 10 *μ*m. The relative ratio of the growth cone area was measured and is shown in the right panel. Mean ± SEM, *n* = 4; ^∗^ *P* < 0.05 versus vector control; ^#^ *P* < 0.05 versus indicated group. (b) Representative images of GFP staining from hippocampal neurons with the same transfection as in (a) are shown. Axon length was measured as the mean ± SEM from three independent experiments. ^∗^ *P* < 0.05 versus vector control; ^∗∗^ *P* < 0.01 versus vector control; ^#^ *P* < 0.05 versus indicated group. Scale bar: 100 *μ*m.

Because CRMP2 and CRMP4 can form tetramers and we found that they were associated with the cytoskeleton and work coordinately to regulate axonal development (Figures 4 and 5), we next determined whether CRMP2 and CRMP4 regulate axonal development via the cytoskeleton. CRMP2 interacts with tubulin via the CRMP2 323–381 amino acid region [18]. Thus, the truncated construct CRMP2ΔC322 would not interact with tubulin and can also act as a CRMP4 dominant-negative plasmid to block all endogenous CRMP4 interacting partners (Figure 3(b)). CRMP4 interacts with actin via its conserved central dihydroorotase-like domain in the C-terminal [16]. Thus, CRMP4ΔC471 cannot bind actin, but can function as a CRMP2 dominant-negative plasmid. By using these two additional constructs, we found that CRMP4ΔC471 significantly reduced the growth cone size when cotransfected with CRMP2 and CRMP2ΔC322 suppressed the promoting effect of CRMP4 overexpression on growth cone development. We observed a similar result for axonal elongation: CRMP4ΔC471 reversed the promoting effect of CRMP2, and CRMP2ΔC322 reversed the promoting effect of CRMP4 on hippocampal axonal elongation (Figure 6(b)). These data indicate that CRMP2 regulates axonal development via CRMP4-mediated actin interaction, and CRMP4 regulates axonal development via CRMP2-mediated tubulin interaction.

## 4. Discussion

In this study, we demonstrated the coordinated roles of CRMP2 and CRMP4 in mediating cytoskeleton dynamics to regulate growth cone development and axonal elongation. We found that CRMP2 and CRMP4 both interacted with tubulin and actin. CRMP2 and CRMP4 formed complexes bridging the junctions between microtubules and actin to regulate cytoskeleton dynamics during axonal guidance and elongation.

The interaction of microtubules and actin is clearly fundamental to many cellular processes, such as cell motility, growth cone guidance, and neurite outgrowth [6]. There are two potential mechanistic interactions between microtubules and actin, regulatory and structural [32]. For regulatory interactions, microtubules and actin indirectly regulate one another through their effects on signaling cascades, similar to that for Rho family proteins [33–35]. For structural interactions, the two systems are directly linked, similar to that for tip interacting proteins (+TIPs) [36, 37], spectraplakins [38, 39], or MAPs [40], especially tau protein [41–43]. Our results indicated that both CRMP2 and CRMP4 interacted with tubulin and actin, with CRMP2 and CRMP4 both being localized in the T-zone where microtubules and actin contact one another in developing growth cones. These results suggest that CRMP2 and CRMP4 are structural molecules crosslinking microtubule and actin networks. We also detected CRMP2 and CRMP4 in the precipitated sediments of one another using Co-IP and GST-pulldown assays and revealed the colocalization of CRMP2 and CRMP4 in growth cones, suggesting that they may form complexes to mediate their interactions with the cytoskeleton. CRMPs were reported to form homo- or heterotetramers [29, 30]. Although we did not generate direct evidence for their forming of homo- or heterotetramers in this study, which is worthwhile investigating, we speculate that CRMP2 and CRMP4 form complexes* in vivo* to bridge microtubules and actin and function coordinately to regulate growth cone development and axonal elongation. Consistent with our conclusion, a recent study using CRMP4−/− mice and CRMP4 overexpression revealed that CRMP4 regulates growth cone dynamics through interactions with the actin and microtubule cytoskeleton [44]. Except for CRMP complexes, the relationships between CRMP and other cytoskeleton interacting proteins, such as tau, MAP2, spectraplakins, or +TIPs, remain to be fully elucidated.

CRMP2 is widely reported to play a critical role in axonal development. However, CRMP4 shows different functions in different types of neurons. In embryonic motoneurons from the mutant SOD1 mouse model of amyotrophic lateral sclerosis, CRMP4a is upregulated to promote axonal degeneration [45]. In chick dorsal root ganglion neurons, CRMP4b was identified as a convergent regulator of axon inhibition [46] and lies downstream of glycogen synthase kinase 3*β* (GSK-3*β*) [47]. In cortical neurons, forced expression of CRMP4b enhances elongation and branching of neurites [48]. In hippocampal neurons, overexpression of CRMP4 induces a significant increase in axon length [31]. Our results showed that CRMP4 genetic knockdown reduced, whereas CRMP4 overexpression increased, growth cone size and axon length, and these effects are mediated by the coordination with CRMP2 to interaction with the cytoskeleton. Thus, the role of CRMP4 is controversial, and this may be due to the different roles of the various CRMP4 isoforms and their different functions in neurite growth and neuronal regeneration, all of which require further investigation.

CRMPs, except CRMP5, share high similarity in protein sequence homology. Multiple posttranscriptional modifications, such as phosphorylation, reportedly regulate CRMPs. CRMPs can be phosphorylated by a variety of kinases, most notably GSK-3*β* [10], cyclin-dependent kinase 5 (Cdk5) [49], and Rho-associated kinase [50]. Cdk5 serves as the priming kinase for GSK-3 [31]. With the exception of CRMP3, all members of CRMPs contain a Cdk5 consensus phosphorylation site. The phosphorylated status of CRMP influences its affinity with the cytoskeleton. Once phosphorylated, CRMPs no longer interact with tubulin; their microtubule assembly effects are abolished, and neurite outgrowth is inhibited [51]. Although CRMPs, regardless of their phosphorylation state, localize with F-actin structures [51], it remains to be clarified whether their effect on actin dynamics is affected by phosphorylation. How the upstream regulators of CRMPs affect cytoskeleton dynamics also awaits elucidation.

Growth cone guidance and axonal development are critical for the accurate establishment of neuronal circuits, and axonal deficits are seen in many neurological diseases, such as Parkinson's disease and Alzheimer's disease (AD) [52–55]. In particular, hyperphosphorylated CRMP2, at the GSK-3 and Cdk5 phosphorylation sites, has been observed in animal models of AD and human AD cortex [56–58]. As previously mentioned, phosphorylated CRMP2 dissociates from microtubules, losing contact with cytoskeleton [51], blocking axonal trafficking [59], and leading to further axonal degeneration. This phosphorylation inactivation of CRMP2 in patients with AD further promotes the formation of neurofibrillary tangles and neuritic plaques [60]. Thus, drugs to activate CRMPs or to stabilize the cytoskeleton may be potential therapeutics for neurodegenerative diseases [61, 62].

**Figure 7:**
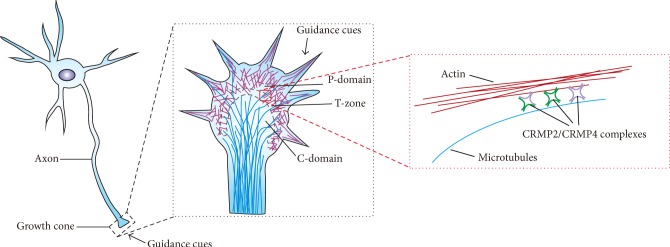
Diagram of a model for CRMP2 and CRMP4 coordinately regulating growth cone development and axon elongation via cytoskeleton dynamics. The schematic model shows that CRMP2 and CRMP4 may form complexes (homo- or heterotetramers) bridging microtubules and actin to mediate cytoskeleton dynamics, thus regulating growth cone development and axon elongation.

In summary, the present study determines that CRMP2 and CRMP4 form complexes that bridge microtubules and actin to mediate the coordinated movement of the cytoskeleton and regulate growth cone development and axonal elongation (Figure 7). These findings provide new insights for the understanding of brain development and therapeutic targets for CRMP-related neurodevelopmental diseases.

## 5. Conclusion

Cytoskeleton dynamics are critical for growth cone development and axonal elongation and guidance. Evidence in this study showed that CRMP2 and CRMP4 formed complexes to interact with microtubules and actin and were colocalized with the cytoskeleton in the T-zone of developing growth cones. CRMP2 and CRMP4 work coordinately, via the interaction with the cytoskeleton, to regulate growth cone development and axon elongation.

## Abbreviations

CRMP: Collapsin response mediator protein
MAP: Microtubule-associated protein
DIV: Days* in vitro*
NC: Negative control
T-zone: Transition-zone
C-domain: Central domain
GSK-3*β*: Glycogen synthase kinase 3*β*
Cdk5: Cyclin-dependent kinase 5.
